# Muscle Strength Is Associated With Physical Function in Community-Dwelling Older Adults Receiving Home Care. A Cross-Sectional Study

**DOI:** 10.3389/fpubh.2022.856632

**Published:** 2022-04-25

**Authors:** Hilde Bremseth Bårdstu, Vidar Andersen, Marius Steiro Fimland, Truls Raastad, Atle Hole Saeterbakken

**Affiliations:** ^1^Department of Sport, Food and Natural Sciences, Faculty of Education, Arts and Sports, Western Norway University of Applied Sciences, Sogndal, Norway; ^2^Department of Neuromedicine and Movement Science, Faculty of Medicine and Health Sciences, Norwegian University of Science and Technology, Trondheim, Norway; ^3^Unicare Helsefort Rehabilitation Centre, Rissa, Norway; ^4^Department of Physical Performance, Norwegian School of Sport Sciences, Oslo, Norway

**Keywords:** elderly, functional ability, independent living, muscular force, home healthcare services

## Abstract

**Background:**

Higher maximal- and explosive strength is associated with better physical function among older adults. Although the relationship between isometric maximal strength and physical function has been examined, few studies have included measures of isometric rate of force development (RFD) as a measure of explosive strength. Furthermore, little is known about the oldest old (>80 years), especially individuals who receive home care and use mobility devices. Therefore, the aim of this study was to examine the association between maximal- and explosive muscle strength with physical function in community-dwelling older adults receiving home care.

**Methods:**

An exploratory cross-sectional analysis including 107 (63 females and 43 males) community-dwelling older adults [median age 86 (interquartile range 80–90) years] receiving home care was conducted. Physical function was measured with five times sit-to-stand (5TSTS), timed 8-feet-up-and-go (TUG-8ft), preferred-, and maximal gait speed. Maximal strength was assessed as maximal isometric voluntary contraction (MVC) and explosive strength as RFD of the knee extensors. We used linear regression to examine the associations, with physical function as dependent variables and muscle strength (MVC and RFD) as independent variables.

**Results:**

MVC was significantly associated with 5TSST [standardized regression coefficient β = −0.26 95% CI (−0.45, −0.06)], TUG-8ft [−0.6 (−0.54, −0.17)], preferred gait speed [0.39 (0.22, 0.57)], and maximal gait speed [0.45 (0.27, 0.62)]. RFD was significantly associated with 5TSST [−0.35 (−0.54, −0.17)], TUG-8ft [−0.43 (−0.60, −0.27)], preferred gait speed [0.40 (0.22, 0.57)], and maximal gait speed [0.48 (0.31, 0.66)].

**Conclusions:**

Higher maximal- and explosive muscle strength was associated with better physical function in older adults receiving home care. Thus, maintaining and/or improving muscle strength is important for perseverance of physical function into old age and should be a priority.

## Introduction

Increasing age is accompanied by a gradual decline in muscle strength (1) which may be explained by reduced muscle mass (e.g., loss of muscle fibers and reduced size of muscle fibers, especially type II fibers), inactivity, and neural factors (e.g., loss of motor neurons) (2, 3). This age-related loss of muscle strength may impair older adults' physical function (e.g., ability to walk, rise from a chair) (3, 4). The reduction of muscle strength and physical function are important components in both sarcopenia and frailty (5, 6) and increases the risk of dependency, institutionalization, and mortality among older adults (5–8). Thus, assessing these aspects is important to develop effective preventive- and treatment strategies especially among the oldest old (>80 years).

Previous cross-sectional studies show that higher muscle strength in the lower body is related to better physical function, such as walking and rising from a chair (9–20). Most previous studies examining the association between muscle strength and physical function have focused on healthy older adults in their 60-s and 70-s (9, 12, 14–18, 20, 21). However, as life expectancy increases worldwide, many live into their 80-s and 90-s and the age-related physiological changes affecting muscle strength and physical function become more prominent after the age of 80 years (1). Consequently, a higher proportion of the aging population will depend on mobility devices (e.g., rollator, walker, canes) and home care services (22, 23). Despite this, only a handful of studies have examined the oldest old (>80 years) (10, 11, 13, 19) and these studies are limited to healthy older adults (10, 19) and/or institutionalized participants able to walk independently (11, 13). Use of mobility devices could influence both muscle strength and physical function as such devices may compensate for lower extremity weakness and loss of mobility (24). This leaves a gap in the literature, and it is important to examine the association between muscle strength and physical function among very old (>80 years) frail individuals who receive home care services, where the need for mobility devices might be high (22, 23).

Most studies examining the association between muscle strength and physical function have measured muscle strength dynamically, especially explosive strength [i.e., power (force × velocity)] (11–13, 15, 16, 18–21, 25). However, evaluating dynamic strength can be challenging for older adults, as it may require high technical skills, sufficient balance and coordination, proper equipment and familiarization, and multiple attempts (26, 27). These challenges become even more apparent for the oldest old (>80 years) and/or those who depend on mobility devices. A possible alternative to overcome the abovementioned challenges is to measure muscle strength isometrically. This enables measurement of maximal strength as maximal voluntary contraction (MVC) and explosive strength as isometric rate of force development (RFD) with high level of control, making it safe, easy, and practical to perform for older adults (26, 27). Despite this, few studies on the oldest old (>80 years) have used isometric measures for muscle strength (13). Furthermore, RFD which is obtained from the slope of the force-time curve (Δforce/Δtime), has been proposed as an important determinant for daily life activities, maintaining postural balance, and avoiding falls among older adults (27–29). Although a few cross-sectional studies have shown that higher RFD is associated with better physical function in 60- and 70-year-olds (9, 14, 15, 17), more research is needed to understand the relationship between RFD and physical function, especially among the oldest old (>80 years). However, to our knowledge, this has not been reported in the existing literature. Finally, although studies indicate that explosive strength is more important for physical function than maximal strength (25), there is a lack of studies including the oldest old and examining explosive strength (i.e., isometric RFD). Thus, the aim of this cross-sectional study was to investigate the association between maximal- and explosive strength with physical function among very old community-dwelling individuals receiving home care.

## Materials and Methods

### Study Design

This exploratory paper used cross-sectional baseline data from a cluster randomized controlled trial (RCT) conducted in three Norwegian municipalities (Sogndal, Luster, and Leikanger) in the period 2016–2019 (trial registration ISRCTN registry 1067873). The RCT was evaluated by The Regional Committee for Medical and Health Research Ethics South-East and the Norwegian Centre for Research Data (2016/51 and 49361/s/AGH, respectively), and was conducted in accordance with the Declaration of Helsinki and Norwegian laws and regulations. Participants received oral and written information about the study before signing a written consent-form. The results from the RCT have been published previously (30, 31).

### Participants

The health care services in the three included municipalities identified potential participants. We used a convenience sample strategy, thus, all inhabitants in Sogndal, Luster, and Leikanger who fulfilled the inclusion/exclusion criteria were invited to participate in the study. We included those who were above 70 years old, community-dwelling, and received home care due to functional and/or medical disabilities. The exclusion criteria included serious cognitive impairments (e.g., Alzheimer's disease, dementia), diagnoses/conditions hindering testing or training, or disapproval from a medical doctor due to contraindications. We made an amendment to the inclusion criteria during participant recruitment; seven older adults otherwise meeting the eligibility criteria, but who were below 70 years [median age 67 (range 63–69) years] were included in the study to increase the sample size.

All inhabitants in the three municipalities who met the inclusion criteria were invited to participate in the study, and all those who accepted were included. Based on this, 123 older adults were initially invited to participate, and six individuals were invited after the initial recruitment. Of these, 19 declined to participate and three participants who were in a wheelchair were excluded as they could not perform testing and/or training. The final sample consisted of 107 participants (Table 1).

**Table 1:** Participant's characteristics.

	***N***	**Males^b^**	**Females^c^**	**Total**
Age (years), median (IQR)	107	85 (80–90)	87 (81–90)	86 (80–90)
Mobility devices, *n* (%)^a^	104	27 (68)	35 (55)	62 (60)
Body Mass Index (kg/m^2^), mean (SD)	103	27 (5)	26 (6)	27 (6)
5TSTS (s), mean (SD)	105	20.4 (8.4)	19.6 (10.6)	19.9 (9.7)
TUG-8ft (s), mean (SD)	103	16.0 (7.4)	14.7 (7.5)	15.2 (7.4)
Preferred gait speed (m/s), mean (SD)	104	0.7 (0.3)	0.8 (0.2)	0.7 (0.3)
Maximal gait speed (m/s), mean (SD)	104	1.0 (0.4)	1.0 (0.3)	1.0 (0.4)
Absolute MVC (N), mean (SD)	105	212.8 (92.6)	160.1 (53.8)	181.2 (76.0)
Relative MVC (N/kg), mean (SD)	101	2.7 (1.2)	2.5 (0.8)	2.6 (0.9)
Absolute RFD (N/s), mean (SD)	105	525.9 (385.6)	353.5 (215.0)	422.5 (305.6)
Relative RFD (N/s/kg), mean (SD)	101	6.7 (4.8)	5.4 (3.8)	5.9 (3.8)

*SD, standard deviation; IQR, interquartile range; 5TSTS, five times sit-to-stand; TUG-8ft, timed 8-feet-up-and-go; MVC, maximal voluntary isometric contraction; RFD, rate of force development.*

a *Mobility devices include rollator, walker, and cane(s).*

b *43 males in total.*

c *64 females in total*.

### Procedures

Testing was conducted at the health care centers by qualified researchers and research assistants. The participants performed two to three trials depending on their individual physical capacity. Time was measured using a stopwatch. For tests of physical function, participants were allowed to use mobility devices and/or the handrails of the chair if necessary. Participants' age and gender was registered, and height was measured using a stadiometer. Body mass was measured in light clothes using a Tanita weight (Tanita MC 780MA S, Illinois, USA) and body mass index (BMI) was calculated as kg/m^2^.

#### Dependent Variables

The ability to rise from a chair was measured as the time taken to finish five sit-to-stand cycles (5TSTS) as fast as possible (32). A straight back chair with armrests was used and participants were told to fully extend their legs in the upright position. The best trial was used for analyses. The 5TSTS test has shown high reliability with Intraclass Correlation Coefficients (ICCs) ranging from 0.64 to 0.96 (33).

For timed 8-feet-up-and-go (TUG-8ft) the participants were instructed to rise from a chair, walk 8 feet, turn around a cone, and walk back to the chair and sit down. The test was performed in a fast, but controlled manner (34). A straight back chair with armrests was used and the best trial was included in the analyses. An ICC of 0.79 has been reported for TUG-8ft (35).

To assess preferred- and maximal gait speed, participants walked a 20-m course (i) in their comfortable pace and (ii) as fast as possible without running (36). A one-meter acceleration- and retardation phase was included before and after the 20-meter course. For preferred gait speed we included the mean of three trials in the analyses, while for maximal gait speed the best trial was used. An ICC of ≥0.903 has been reported for preferred- and maximal 10-m gait speed (37).

#### Independent Variables

**Figure 1:**
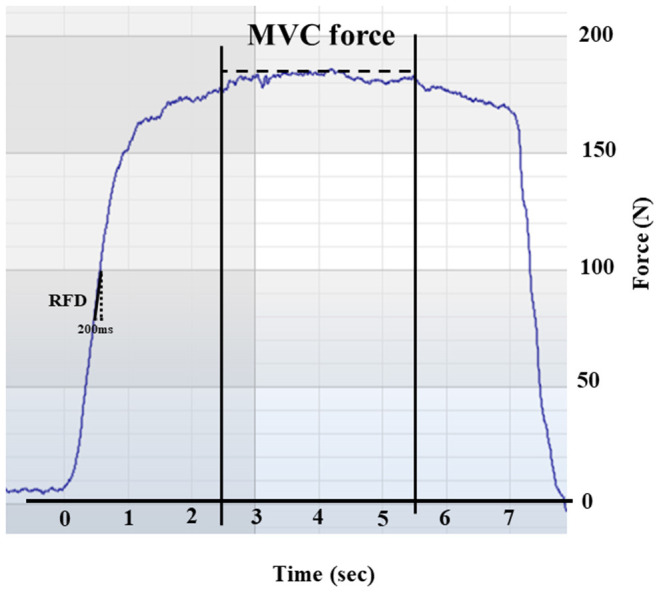
Representative force-time curve obtained during a maximal isometric voluntary contraction (MVC) in a single subject. The figure illustrates the rate of force development (RFD) calculated over a 200-ms window and the MVC calculated over a 300-s window.

Muscle strength was measured during a maximal voluntary isometric contraction (MVC) of the knee extensors. A custom-made flexi-bench (Pivot 430 Flexi-bench, Sportsmaster, Norway) and a non-elastic band (ROPES A/S, Aasgardstrand, Norway) attached to a force cell (Ergotest Innovation AS, Langesund, Norway) was used. We used a frequency of 200 Hz and a range of 0–500 kg. The knee was fixed at a 90-degree angle and the band was placed around the preferred ankle. Participants were told to contract as “fast and forcefully” as possible for at least 5 s, with a 1-min resting period between trials. The best trial was used in analyses. As all the dependent variables were weight bearing, we calculated relative maximal- and explosive muscle strength (normalized to body mass). Maximal strength (i.e., MVC) was defined as the highest mean force output over a 3-second window. Explosive strength (i.e., RFD) was calculated at the steepest vertical force generation as the mean tangential slope of the force-time curve over a 200-ms window (see Figure 1 for a typical example of a force-time curve) (38). A 200-ms interval was chosen for analysis because weaker, very old individuals might use a longer time to peak force from the onset of force than younger and/or stronger individuals (9, 27). Furthermore, we took into consideration our previous experience from a pilot study (39) when it comes to force-time curves, ability to understand the task (e.g., generating force as fast and forcefully as possible), and fear of pain and/or movement in this particular group of older adults, when choosing the window length. The correlation between MVC and RFD was *r* = 0.67.

### Statistical Analysis

Demographic participant characteristics are presented as mean and SD or median and IQR. To assess normality, the Q-Q plots of the residuals were visually inspected. The associations were examined using linear regression with the continuous variables of physical function as dependent variables and the muscle strength measures (MVC and RFD) as independent variables. We conducted analyses for each combination of physical function- and muscle strength measure. Due to some extreme values, we performed sensitivity analyses without extreme values to assess the robustness of our results. Visual inspection of the entire data set was used to assess these extreme values. All analyses were adjusted for gender (40). Standardized beta coefficients (ß) and 95% confidence intervals (CI) was calculated to show the strength of the independent variable to the dependent variable. A *p*-value ≤ 0.05 was defined as statistically significant.

All analyses were conducted in STATA 16 (StataCorp. 2019. *Stata Statistical Software: Release 16*. College Station, TX: StataCorp LLC) and Supplementary Figures 1–4 were made in SigmaPlot 14.0 (Systat Software, Inc., San Jose, CA, USA).

## Results

Baseline characteristics of the participants are presented in Table 1. The sample consisted of 64 females (60%, body mass 65.5 kg, height 157 cm) and 43 males (40%, body mass 77.4 kg, height 169 cm). The males were slightly younger (85 vs. 87 years), stronger, and had a higher percentage of mobility device usage (68 vs. 55%) than the women. Data for physical function were available in 97–98% of participants, while data on MVC and RFD was available in 94% of participants. The number of participants included in the analyses ranged from 99 to 100.

### Associations Between Muscle Strength and Physical Function

The regression analyses showed that both MVC and RFD were significantly associated with all physical function measures (*p* < 0.01 for all). For MVC there were negative (favorable) associations with 5TSTS [β = −0.26 95% CI (−0.45, −0.06)] and TUG-8ft [−0.36 (−0.53, −0.19)], and positive (favorable) associations with preferred- [0.39 (0.22, 0.57)] and maximal gait speed [0.45 (0.27, 0.62)]. For RFD there were negative (favorable) associations with 5TSTS [−0.35 (−0.54, −0.17)] and TUG-8ft [−0.43 (−0.60, −0.27)], and positive (favorable) associations with preferred- [0.40 (0.22, 0.57)] and maximal gait speed [0.48 (0.31, 0.66)]. Supplementary Table S1 show the unstandardized regression coefficients.

### Sensitivity Analysis

Supplementary Table S2 show the results from the sensitivity analysis after removing extreme values. The number of participants analyzed ranged from 93 to 96. MVC was associated with 5TSST [β = −0.40 95% CI (−0.59, −0.21)], TUG-8ft [−0.39 (−0.81, −0.21)], preferred- [0.35 (0.18, 0.53)], and maximal gait speed [0.42 (0.24, 0.59)]. RFD was associated with 5TSST [−0.29 (−0.49, −0.09)], TUG-8ft [−0.43 (−0.61, −0.24)], preferred- [0.36 (0.18, 0.53)], and maximal gait speed [0.47 (0.30, 0.64)].

## Discussion

This cross-sectional study showed that higher maximal- and explosive strength were associated with better physical function in the oldest old who receive home care. These findings suggest that maintaining and/or improving muscle strength is important for perseverance of physical function into old age.

Some previous cross-sectional studies have investigated the relationship between muscle strength and physical function among the oldest old (>80 years) (10, 11, 13, 19). Barbat-Artigas et al. (10) showed that ambulatory women (mean age 80.4 years) in the lowest maximal leg-strength quartile was 12–25-fold more likely to have impairments in chair rise, preferred-, and maximal gait speed compared to those in the highest strength quartile. Likewise, Bassey et al. (11) found that explosive strength, measured as leg extension power, was related to chair rise, stair climb, and gait speed (*r* = 0.65–0.81) in residents of a rehabilitation center where 65% used mobility devices. These previous findings are in line with ours, however, direct comparisons between studies are difficult due to the focus on slightly different populations and aspects of physical function. Moreover, explosive strength has in previous studies been assessed by dynamic measures (i.e., power), especially among the oldest old (11, 19). Although Altubasi (9) showed that higher isometric rate of torque development (RTD) was moderately correlated with stair climb time (*r* = −0.59), correlations were weak for TUG, ramp up, and preferred gait speed (*r* = −0.12 to −0.29) in healthy older adults in their 60s and 70s. Similarly, Osawa et al. (17) found that RTD was important for some, but not all, measures of physical function among healthy older adults in their 60s. However, these results might not be entirely comparable to ours as the relationship between muscle strength and physical function is believed to be curvilinear, creating a threshold where muscle strength is less important for physical function, especially in younger, stronger older adults (12, 41). Thus, our results support those of previous studies showing that higher muscle strength is associated with better physical function in the oldest old and expand on the existing literature by including individuals who receive home care and with high mobility device dependency (60%), which is an important and increasing group of older adults.

Explosive strength has been found to be more important for physical function than maximal strength among older adults in their 60-s and 70-s (12, 18, 42). Although evidence suggest that explosive strength decreases more rapidly than maximal strength with increasing age (2) few of the previous studies have examined the oldest old (>80 years) have included measures of both maximal- and explosive strength (13). To indicate the strength of the associations, we calculated standardized regression coefficients which indicated a slightly stronger association for explosive strength (RFD) than maximal strength (MVC) with all measures of physical function. Rising from a chair as fast as possible involves repetitive acceleration of one's body mass and may demand less time to develop force and a higher level of explosive- than maximal strength (43). Similarly, the acceleration of body mass is also relevant for TUG-8ft performance and walking as fast as possible. It should be mentioned that the 95% CIs for the standardized regression coeffects overlap substantially, making it difficult to draw inferences regarding the importance of maximal- vs. explosive strength from our results. Furthermore, we used a 200-ms window to assess RFD, and RFD measured during the later phase of rising muscle force has been found to be closely related to MVC (44). Thus, there is most likely a relation between the two measures. However, a stronger association for explosive strength can be supported by the age-related degeneration in the muscle (e.g., atrophy of type II fibers, cross-sectional area, fewer motor units, and reduced motor unit firing rate) (45). We cannot exclude the possibility that some extreme values affected our findings. Therefore, we performed a sensitivity analysis to assess the influence of extreme values showing no major changes in the standardized regression coefficients. However, it should be noted that the standardized regression coefficient for MVC with 5TSTS increased from −0.26 to −0.40, possibly indicating that maximal strength is even more important for the ability to rise from a chair than initially found.

The progressive atrophy of muscle fibers reported with increasing age is greater for type II muscle fibers than for type I muscle fibers (46). Type II muscle fibers are especially important during fast movements and, consequently, explosive strength might be more impaired than maximal strength (27, 38). Resistance training using maximal intentional acceleration of load (i.e., explosive type) has shown superior effects on explosive strength and physical function when compared to traditional heavy load resistance training (47). However, heavy loads resistance training has shown to increase the size of type II muscle fibers and myosin heavy chain II A proportion in 85–97-year-olds (48), which might be effective for eliciting gains in explosive strength (27). Thus, designing heavy loads resistance training programs with maximal intentional acceleration of the load (“explosive heavy load type”) (38, 48) could be the optimal combination for improving older adults' explosive strength, and consequently maintaining or improving physical function in old age. Additionally, such a training program would be beneficial for increasing maximal strength as well.

Isometric testing of older adults' muscle strength holds several advantages, as it requires less technical skills, balance, and coordination than dynamic strength testing (26, 27). Furthermore, isometric testing enables a high level of control, making it safe, easy, and practical to perform (26, 27). Although dynamic power has been measured previously in the oldest old during chair rise (11) and a facilitated jump test (19), these tests require higher technical skills and can be difficult to perform for older adults, especially for those who depend on mobility devices. Furthermore, many daily life movements (e.g., rapid walking, postural balance, preventing a fall) require rapid force production over a short time frame (e.g., 50–300 ms) (27–29). As RFD can be obtained from the force-time curve (27) it is a relevant measure of older adults' explosive strength. Thus, the present study show that isometric testing is a viable, practical, and safe alternative for assessment of muscle strength in older adults, also when the proportion of mobility device use is high.

Previous studies have suggested that the relationship between muscle strength and physical function is curvilinear, creating a threshold above which an increase in strength does not translate into improved physical function (12, 41, 49, 50). Identification of a specific threshold would be useful to target those with an increased risk of functional limitations who would most likely benefit from resistance training. We did not aim to statistically investigate non-linearity. Furthermore, our participants were very old with poor muscle strength and physical function (e.g., 60% used mobility devices), and identification of a clear threshold may not be possible in such a population (41, 49, 50). Nevertheless, visual inspection of the strength-function curves indicated that if a threshold (i.e., point of change in slope) exist, it is at the far range of our data, around 5.6–6.2 N/kg and 14.1–16.7 N/s/kg for MVC and RFD, respectively (Supplementary Figures 1–4). Importantly, there are very few data points above this, thus, the observed threshold may be due to random variation and should be interpreted with caution.

Reference estimates of older adults' physical function are often derived from apparently healthy populations (32, 51, 52), which excludes more frail individuals. However, as life expectancy increases, many older adults will live into their 80-s and 90-s, and many will be dependent on home care and mobility devices to function in their own home. Thus, healthy, younger older adults are not representative for the entire older population. In the present study, the participants were classified as the oldest old, all received home care, and 60% used mobility devices. Accordingly, their physical function was in line with or slightly lower than those reported by Lusardi et al. (22) for older adults (80–101 years) with- and without mobility devices. Furthermore, the maximal strength was low and comparable to those shown by Aas et al. (53) in a comparable sample, although direct comparison is difficult due to different methods used to assess maximal strength. Thus, our findings highlight the importance of obtaining knowledge about the level of, and association between, muscle strength and physical function in this rapidly growing group of older adults, and not only in younger, healthier, and more well-functioning individuals.

The strengths of our study include the choice of participants (i.e., oldest old, receiving home care, mobility devices) which allows for knowledge about an understudied, yet important group of older adults. Furthermore, we examined both maximal- and explosive muscle strength, and used isometric measures to assess muscle strength. Some study limitations should be addressed. First, this was an exploratory study and the cross-sectional design precludes determination of the temporal relationship between muscle strength and physical function, as well as causality. Second, the study may not have been powered to investigate the associations included in the current paper. Third, our data material showed large SDs and some extreme values. This was not surprising given the variation in age, strength, and functional status seen among older adults receiving home care. It may be that the differences in muscle strength between the genders influenced the distribution of the data, and hence, the results. However, we did use relative muscle strength which may take some of the gender differences into account. Fourth, we did not investigate whether the association between muscle strength and physical function differed according to use of mobility devices, as introducing mobility devices as a covariate in this regression analysis would introduce a collider bias (54). Future studies should examine the impact of mobility devices on the association between muscle strength and physical function. Lastly, although we included measures of both maximal- and explosive strength our analyses did not investigate their independent contributions, which should be examined in future studies. Based on the abovementioned limitations we advise reflective interpretation of the results.

In conclusion, the present study shows that higher maximal- and explosive muscle strength is associated with better physical function in the oldest old who receive home care. Our findings add knowledge about a rapidly growing yet understudied group of older adults and highlight the importance of prioritizing strategies aiming to maintain and/or improve muscle strength for perseverance of physical function into old age.

## Data Availability Statement

The raw data supporting the conclusions of this article will be made available by the authors, without undue reservation.

## Ethics Statement

The studies involving human participants were reviewed and approved by the Regional Committee for Medical and Health Research Ethics (2016/51), Armauer Hansens Hus, nordre fløyel, 2. etasje, Haukelandsveien 28, Bergen; Norwegian Centre for Research Data (49361/s/AGH), Harald Hårfagres gate 29 N-5007, Bergen, Norway. The patients/participants provided their written informed consent to participate in this study.

## Author Contributions

HB oversaw the main writing of the manuscript and data analyses. AS, VA, MF, and TR contributed to planning the study, while AS and VA were in charge of running the study and collected data. AS, VA, MF, and TR reviewed the manuscript and gave valued input on revisions. All authors read and approved the final manuscript.

## Funding

This study was funded by the Norwegian Regional Research Council of Western Norway (RFV, Project Number: 257071).

## Conflict of Interest

The authors declare that the research was conducted in the absence of any commercial or financial relationships that could be construed as a potential conflict of interest.

## Publisher's Note

All claims expressed in this article are solely those of the authors and do not necessarily represent those of their affiliated organizations, or those of the publisher, the editors and the reviewers. Any product that may be evaluated in this article, or claim that may be made by its manufacturer, is not guaranteed or endorsed by the publisher.

## References

[1] Frontera WR Hughes VA Fielding RA Fiatarone MA Evans WJ Roubenoff R. Aging of skeletal muscle: a 12-yr longitudinal study. J Appl Physiol Bethesda Md 1985. (2000) 88:1321–6. 10.1152/jappl.2000.88.4.1321 10749826

[2] Aagaard P Suetta C Caserotti P Magnusson SP Kjaer M. Role of the nervous system in sarcopenia and muscle atrophy with aging: strength training as a countermeasure. Scand J Med Sci Sports. (2010) 20:49–64. 10.1111/j.1600-0838.2009.01084.x 20487503

[3] American American College of Sports Medicine Chodzko-Zajko WJ Proctor DN Fiatarone Singh MA Minson CT Nigg CR. American College of Sports Medicine position stand. Exercise and physical activity for older adults. Med Sci Sports Exerc. (2009) 41:1510–30. 10.1249/MSS.0b013e3181a0c95c 19516148

[4] Fragala MS Cadore EL Dorgo S Izquierdo M Kraemer WJ Peterson MD. Resistance training for older adults: position statement from the national strength and conditioning association. J Strength Cond Res. (2019) 33:2019–52. 10.1519/JSC.0000000000003230 31343601

[5] Cruz-Jentoft AJ Bahat G Bauer J Boirie Y Bruyère O Cederholm T. Sarcopenia: revised European consensus on definition and diagnosis. Age Ageing. (2019) 48:16–31. 10.1093/ageing/afz046 30312372 PMC6322506

[6] Dodds R Sayer AA. Sarcopenia and frailty: new challenges for clinical practice. Clin Med. (2016) 16:455–8. 10.7861/clinmedicine.16-5-455 27697810 PMC6297299

[7] Wang DXM Yao J Zirek Y Reijnierse EM Maier AB. Muscle mass, strength, and physical performance predicting activities of daily living: a meta-analysis. J Cachexia Sarcopenia Muscle. (2020) 11:3–25. 10.1002/jcsm.12502 31788969 PMC7015244

[8] Akune T Muraki S Oka H Tanaka S Kawaguchi H Tokimura F. Incidence of certified need of care in the long-term care insurance system and its risk factors in the elderly of Japanese population-based cohorts: the ROAD study. Geriatr Gerontol Int. (2014) 14:695–701. 10.1111/ggi.12155 24020635

[9] Altubasi IM. Is quadriceps muscle strength a determinant of the physical function of the elderly? J Phys Ther Sci. (2015) 27:3035–8. 10.1589/jpts.27.3035 26644638 PMC4668129

[10] Barbat-Artigas S Rolland Y Cesari M Abellan van Kan G Vellas B Aubertin-Leheudre M. Clinical relevance of different muscle strength indexes and functional impairment in women aged 75 years and older. J Gerontol Ser A. (2013) 68:811–9. 10.1093/gerona/gls254 23262030

[11] Bassey EJ Fiatarone MA O'Neill EF Kelly M Evans WJ Lipsitz LA. Leg extensor power and functional performance in very old men and women. Clin Sci Lond Engl. (1992) 82:321–7. 10.1042/cs0820321 1312417

[12] Bean JF Kiely DK Herman S Leveille SG Mizer K Frontera WR. The relationship between leg power and physical performance in mobility-limited older people. J Am Geriatr Soc. (2002) 50:461–7. 10.1046/j.1532-5415.2002.50111.x 11943041

[13] Casas-Herrero A Cadore EL Zambom-Ferraresi F Idoate F Millor N Martínez-Ramirez A. Functional capacity, muscle fat infiltration, power output, and cognitive impairment in institutionalized frail oldest old. Rejuvenation Res. (2013) 16:396–403. 10.1089/rej.2013.1438 23822577 PMC3804230

[14] Clark DJ Manini TM Fielding RA Patten C. Neuromuscular determinants of maximum walking speed in well-functioning older adults. Exp Gerontol. (2013) 48:358–63. 10.1016/j.exger.2013.01.010 23376102 PMC3594593

[15] Crockett K Ardell K Hermanson M Penner A Lanovaz J Farthing J. The relationship of knee-extensor strength and rate of torque development to sit-to-stand performance in older adults. Physiother Can. (2013) 65:229–35. 10.3138/ptc.2012-04 24403691 PMC3740986

[16] Cuoco A Callahan DM Sayers S Frontera WR Bean J Fielding RA. Impact of muscle power and force on gait speed in disabled older men and women. J Gerontol A Biol Sci Med Sci. (2004) 59:1200–6. 10.1093/gerona/59.11.1200 15602076

[17] Osawa Y Studenski SA Ferrucci L. Knee extension rate of torque development and peak torque: associations with lower extremity function. J Cachexia Sarcopenia Muscle. (2018) 9:530–9. 10.1002/jcsm.12285 29569834 PMC5989739

[18] Puthoff ML Nielsen DH. Relationships among impairments in lower-extremity strength and power, functional limitations, and disability in older adults. Phys Ther. (2007) 87:1334–47. 10.2522/ptj.20060176 17684086

[19] Rantanen T Avela J. Leg extension power and walking speed in very old people living independently. J Gerontol A Biol Sci Med Sci. (1997) 52:M225–31. 10.1093/gerona/52A.4.M225 9224434

[20] Visser M Newman AB Nevitt MC Kritchevsky SB Stamm EB Goodpaster BH. Reexamining the sarcopenia hypothesis. Muscle mass versus muscle strength. Health, Aging, and Body Composition Study Research Group. Ann N Y Acad Sci. (2000) 904:456–61. 10.1111/j.1749-6632.2000.tb06500.x 10865789

[21] McCarthy EK Horvat MA Holtsberg PA Wisenbaker JM. Repeated chair stands as a measure of lower limb strength in sexagenarian women. J Gerontol A Biol Sci Med Sci. (2004) 59:1207–12. 10.1093/gerona/59.11.1207 15602077

[22] Lusardi MM Pellecchia GL Schulman M. Functional performance in community living older adults. J Geriatr Phys Ther. (2003) 26:14–22. 10.1519/00139143-200312000-0000320534778

[23] World Health Organization. Regional Office for the Eastern Mediterranean. The growing need for home health care for the elderly: home health care for the elderly as an integral part of primary health care services. World Health Organization; Regional Office for the Eastern Mediterranean (2015). Available online at: https://applications.emro.who.int/dsaf/EMROPUB_2015_EN_1901.pdf?ua=1

[24] Bradley SM Hernandez CR. Geriatric assistive devices. Am Fam Physician. (2011) 84:405–11. 21842786

[25] Byrne C Faure C Keene DJ Lamb SE. Ageing, muscle power and physical function: a systematic review and implications for pragmatic training interventions. Sports Med Auckl NZ. (2016) 46:1311–32. 10.1007/s40279-016-0489-x 26893098

[26] Drake D Kennedy R Wallace E. The validity and responsiveness of isometric lower body multi-joint tests of muscular strength: a systematic review. Sports Med Open. (2017) 3:23. 10.1186/s40798-017-0091-2 28631257 PMC5476535

[27] Rodríguez-Rosell D Pareja-Blanco F Aagaard P González-Badillo JJ. Physiological and methodological aspects of rate of force development assessment in human skeletal muscle. Clin Physiol Funct Imaging. (2018) 38:743–62. 10.1111/cpf.12495 29266685

[28] Aagaard P. Training-induced changes in neural function. Exerc Sport Sci Rev. (2003) 31:61–7. 10.1097/00003677-200304000-00002 12715968

[29] Aagaard P Simonsen EB Andersen JL Magnusson P Dyhre-Poulsen P. Increased rate of force development and neural drive of human skeletal muscle following resistance training. J Appl Physiol Bethesda Md 1985. (2002) 93:1318–26. 10.1152/japplphysiol.00283.2002 12235031

[30] Bårdstu HB Andersen V Fimland MS Aasdahl L Raastad T Cumming KT. Effectiveness of a resistance training program on physical function, muscle strength, and body composition in community-dwelling older adults receiving home care: a cluster-randomized controlled trial. Eur Rev Aging Phys Act. (2020) 17:11. 10.1186/s11556-020-00245-7 32782626 PMC7414534

[31] Bårdstu HB Andersen V Fimland MS Aasdahl L Lohne-Seiler H Saeterbakken AH. Physical activity level following resistance training in community-dwelling older adults receiving home care: results from a cluster-randomized controlled trial. Int J Environ Res Public Health. (2021) 18:6682. 10.3390/ijerph18136682 34206175 PMC8297335

[32] Bohannon RW. Reference values for the five-repetition sit-to-stand test: a descriptive meta-analysis of data from elders. Percept Mot Skills. (2006) 103:215–22. 10.2466/pms.103.1.215-222 17037663

[33] Bohannon RW. Test-retest reliability of the five-repetition sit-to-stand test: a systematic review of the literature involving adults. J Strength Cond Res. (2011) 25:3205–7. 10.1519/JSC.0b013e318234e59f 21904240

[34] Rikli RE Jones CJ. Development and validation of criterion-referenced clinically relevant fitness standards for maintaining physical independence in later years. The Gerontologist. (2013) 53:255–67. 10.1093/geront/gns071 22613940

[35] Jette AM Jette DU Ng J Plotkin DJ Bach MA. Are performance-based measures sufficiently reliable for use in multicenter trials? Musculoskeletal Impairment (MSI) Study Group. J Gerontol A Biol Sci Med Sci. (1999) 54:M3–6. 10.1093/gerona/54.1.M3 10026655

[36] Motyl JM Driban JB McAdams E Price LL McAlindon TE. Test-retest reliability and sensitivity of the 20-meter walk test among patients with knee osteoarthritis. BMC Musculoskelet Disord. (2013) 14:166. 10.1186/1471-2474-14-166 23663561 PMC3661341

[37] Bohannon RW. Comfortable and maximum walking speed of adults aged 20-79 years: reference values and determinants. Age Ageing. (1997) 26:15–9. 10.1093/ageing/26.1.15 9143432

[38] Caserotti P Aagaard P Larsen JB Puggaard L. Explosive heavy-resistance training in old and very old adults: changes in rapid muscle force, strength and power. Scand J Med Sci Sports. (2008) 18:773–82. 10.1111/j.1600-0838.2007.00732.x 18248533

[39] Saeterbakken AH Bårdstu HB Brudeseth A Andersen V. Effects of strength training on muscle properties, physical function, and physical activity among frail older people: a pilot study. J Aging Res. (2018) 2018:e8916274. 10.1155/2018/8916274 29988285 PMC6008824

[40] Bouchard DR Héroux M Janssen I. Association between muscle mass, leg strength, and fat mass with physical function in older adults: influence of age and sex. J Aging Health. (2011) 23:313–28. 10.1177/0898264310388562 21081704

[41] Buchner DM Larson EB Wagner EH Koepsell TD de Lateur BJ. Evidence for a non-linear relationship between leg strength and gait speed. Age Ageing. (1996) 25:386–91. 10.1093/ageing/25.5.386 8921145

[42] Bean JF Leveille SG Kiely DK Bandinelli S Guralnik JM Ferrucci L. A comparison of leg power and leg strength within the InCHIANTI study: which influences mobility more? J Gerontol A Biol Sci Med Sci. (2003) 58:728–33. 10.1093/gerona/58.8.M728 12902531

[43] Hardy R Cooper R Shah I Harridge S Guralnik J Kuh D. Is chair rise performance a useful measure of leg power? Aging Clin Exp Res. (2010) 22:412–8. 10.1007/BF03324942 21422795 PMC3260651

[44] Andersen LL Aagaard P. Influence of maximal muscle strength and intrinsic muscle contractile properties on contractile rate of force development. Eur J Appl Physiol. (2006) 96:46–52. 10.1007/s00421-005-0070-z 16249918

[45] Piasecki M Ireland A Jones DA McPhee JS. Age-dependent motor unit remodelling in human limb muscles. Biogerontology. (2016) 17:485–96. 10.1007/s10522-015-9627-3 26667009 PMC4889636

[46] Lexell J Taylor CC Sjöström M. What is the cause of the ageing atrophy? Total number, size and proportion of different fiber types studied in whole vastus lateralis muscle from 15- to 83-year-old men. J Neurol Sci. (1988) 84:275–94. 3379447 10.1016/0022-510x(88)90132-3

[47] Sayers SP. High velocity power training in older adults. Curr Aging Sci. (2008) 1:62–7. 10.2174/1874609810801010062 20021374

[48] Kryger AI Andersen JL. Resistance training in the oldest old: consequences for muscle strength, fiber types, fiber size, and MHC isoforms. Scand J Med Sci Sports. (2007) 17:422–30. 10.1111/j.1600-0838.2006.00575.x 17490465

[49] Ferrucci L Guralnik JM Buchner D Kasper J Lamb SE Simonsick EM. Departures from linearity in the relationship between measures of muscular strength and physical performance of the lower extremities: the Women's Health and Aging Study. J Gerontol A Biol Sci Med Sci. (1997) 52:M275–85. 10.1093/gerona/52A.5.M275 9310081

[50] Jette AM Assmann SF Rooks D Harris BA Crawford S. Interrelationships among disablement concepts. J Gerontol A Biol Sci Med Sci. (1998) 53:M395–404. 10.1093/gerona/53A.5.M395 9754147

[51] Bohannon RW. Reference values for the timed up and go test: a descriptive meta-analysis. J Geriatr Phys Ther 2001. (2006) 29:64–8. 10.1519/00139143-200608000-00004 16914068

[52] Fritz S Lusardi M. White paper: “walking speed: the sixth vital sign.” *J Geriatr Phys Ther*. (2009) 32:46–9. 10.1519/00139143-200932020-00002 20039582

[53] Aas SN Breit M Karsrud S Aase OJ Rognlien SH Cumming KT. Musculoskeletal adaptations to strength training in frail elderly: a matter of quantity or quality? J Cachexia Sarcopenia Muscle. (2020) 11:663–77. 10.1002/jcsm.12543 32091670 PMC7296272

[54] Greenland S. Quantifying biases in causal models: classical confounding vs collider-stratification bias. Epidemiology. (2003) 14:300–6. 10.1097/01.EDE.0000042804.12056.6C 12859030

