# Supplementary material for: Muscle Strength Is Associated With Physical Function in Community-Dwelling Older Adults Receiving Home Care. A Cross-Sectional Study
Source: Front Public Health. 2022 Apr 25;10:856632. doi: 10.3389/fpubh.2022.856632 (PMC9081336; doi:10.3389/fpubh.2022.856632)
Supplement: Supplementary file 1 [file Table_1.docx]

Table S1. Regression analyses showing the association between muscle strength and physical function.

|  | *N* | Regression coefficient | | Standardized regression coefficient | | p-value |
| --- | --- | --- | --- | --- | --- | --- |
|  |  | B | 95% CI | ß | 95% CI |  |
| 5TSTS |  |  |  |  |  |  |
| Relative MVC | 100 | -2.63 | -4.59, -0.67 | -0.26 | -0.45, -0.06 | 0.009 |
| Relative RFD | 100 | -0.90 | -1.37, -0.42 | -0.35 | -0.54, -0.17 | <0.001 |
| TUG-8ft |  |  |  |  |  |  |
| Relative MVC | 99 | -2.84 | -4.19, -1.50 | -0.36 | -0.53, -0.19 | <0.001 |
| Relative RFD | 99 | -0.84 | -1.17, -0.52 | -0.43 | -0.60, -0.27 | <0.001 |
| Preferred gait speed |  |  |  |  |  |  |
| Relative MVC | 99 | 0.10 | 0.06, 0.15 | 0.39 | 0.22, 0.57 | <0.001 |
| Relative RFD | 99 | 0.03 | 0.01, 0.04 | 0.40 | 0.22, 0.57 | <0.001 |
| Maximal gait speed |  |  |  |  |  |  |
| Relative MVC | 99 | 0.18 | 0.11, 0.25 | 0.45 | 0.27, 0.62 | <0.001 |
| Relative RFD | 99 | 0.05 | 0.03, 0.07 | 0.48 | 0.31, 0.66 | <0.001 |

5TSTS, five times sit-to-stand; MVC, maximal voluntary isometric contraction; RFD, rate of force development; TUG-8ft, timed 8-feet-up-and-go; CI, confidence interval; B, unstandardized regression coefficient; ß, standardized regression coefficient. Adjusted for gender.
