# Supplementary material for: Muscle Strength Is Associated With Physical Function in Community-Dwelling Older Adults Receiving Home Care. A Cross-Sectional Study
Source: Front Public Health. 2022 Apr 25;10:856632. doi: 10.3389/fpubh.2022.856632 (PMC9081336; doi:10.3389/fpubh.2022.856632)
Supplement: Supplementary file 2 [file Table_2.docx]

Table S2. Sensitivity analysis without extreme values showing the association between muscle strength and physical function.

|  | *N* | Regression coefficient | | Standardized regression coefficient | | p-value |
| --- | --- | --- | --- | --- | --- | --- |
|  |  | B | 95% CI | ß | 95% CI |  |
| 5TSTS |  |  |  |  |  |  |
| Relative MVC | 94 | -3.12 | -4.58, -1.65 | -0.40 | -0.59, -0.21 | <0.001 |
| Relative RFD | 93 | -0.55 | -0.93, -0.17 | -0.29 | -0.49, -0.09 | 0.005 |
| TUG-8ft |  |  |  |  |  |  |
| Relative MVC | 96 | -3.46 | -5.11, -1.81 | -0.39 | -0.58, -0.21 | <0.001 |
| Relative RFD | 94 | -0.92 | -1.31, -0.53 | -0.43 | -0.61, -0.24 | <0.001 |
| Preferred gait speed |  |  |  |  |  |  |
| Relative MVC | 95 | 0.11 | 0.05, 0.17 | 0.35 | 0.18, 0.53 | <0.001 |
| Relative RFD | 93 | 0.03 | 0.01, 0.04 | 0.36 | 0.18, 0.53 | <0.001 |
| Maximal gait speed |  |  |  |  |  |  |
| Relative MVC | 95 | 0.19 | 0.11, 0.28 | 0.42 | 0.24, 0.59 | <0.001 |
| Relative RFD | 93 | 0.05 | 0.03, 0.07 | 0.47 | 0.30, 0.64 | <0.001 |

5TSTS, five times sit-to-stand; MVC, maximal voluntary isometric contraction; RFD, rate of force development; TUG-8ft, timed 8-feet-up-and-go; CI, confidence interval; B, unstandardized regression coefficient; ß, standardized regression coefficient. Adjusted for gender.
