# Supplementary figures and images for: Muscle Strength Is Associated With Physical Function in Community-Dwelling Older Adults Receiving Home Care. A Cross-Sectional Study
Source: Front Public Health. 2022 Apr 25;10:856632. doi: 10.3389/fpubh.2022.856632 (PMC9081336; doi:10.3389/fpubh.2022.856632)

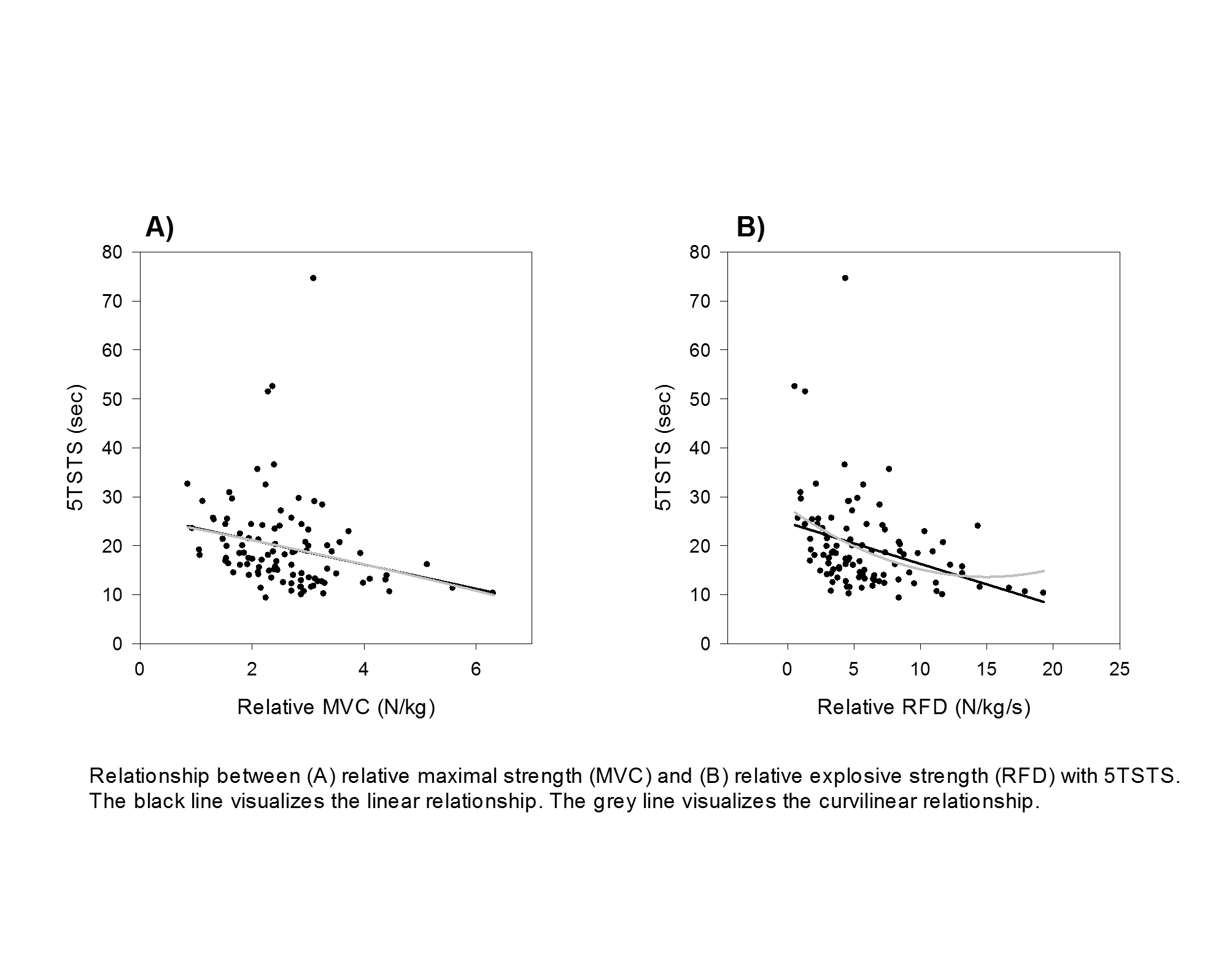

Supplement: Supplementary file 3 [file Image_1.tif]

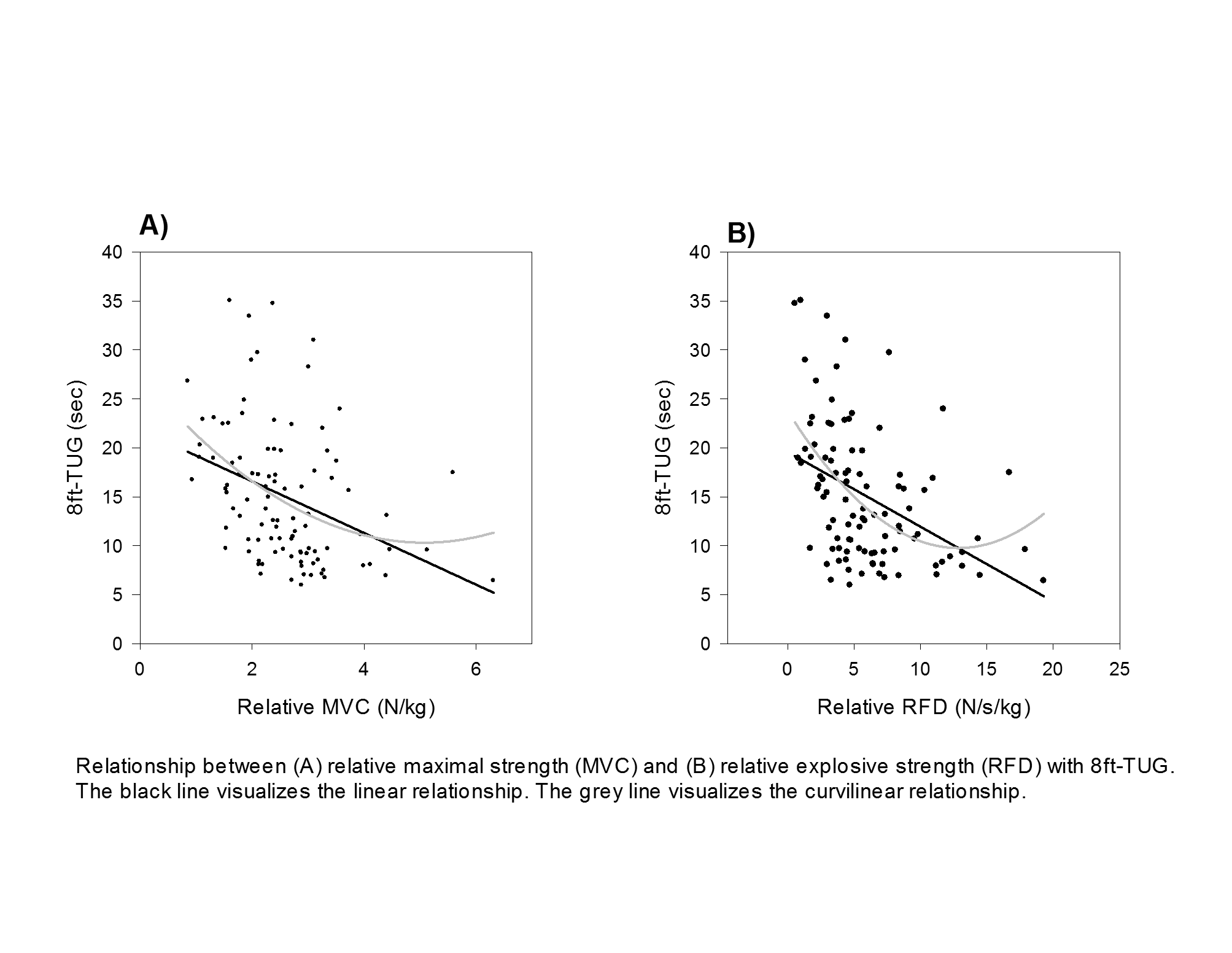

Supplement: Supplementary file 4 [file Image_2.tif]

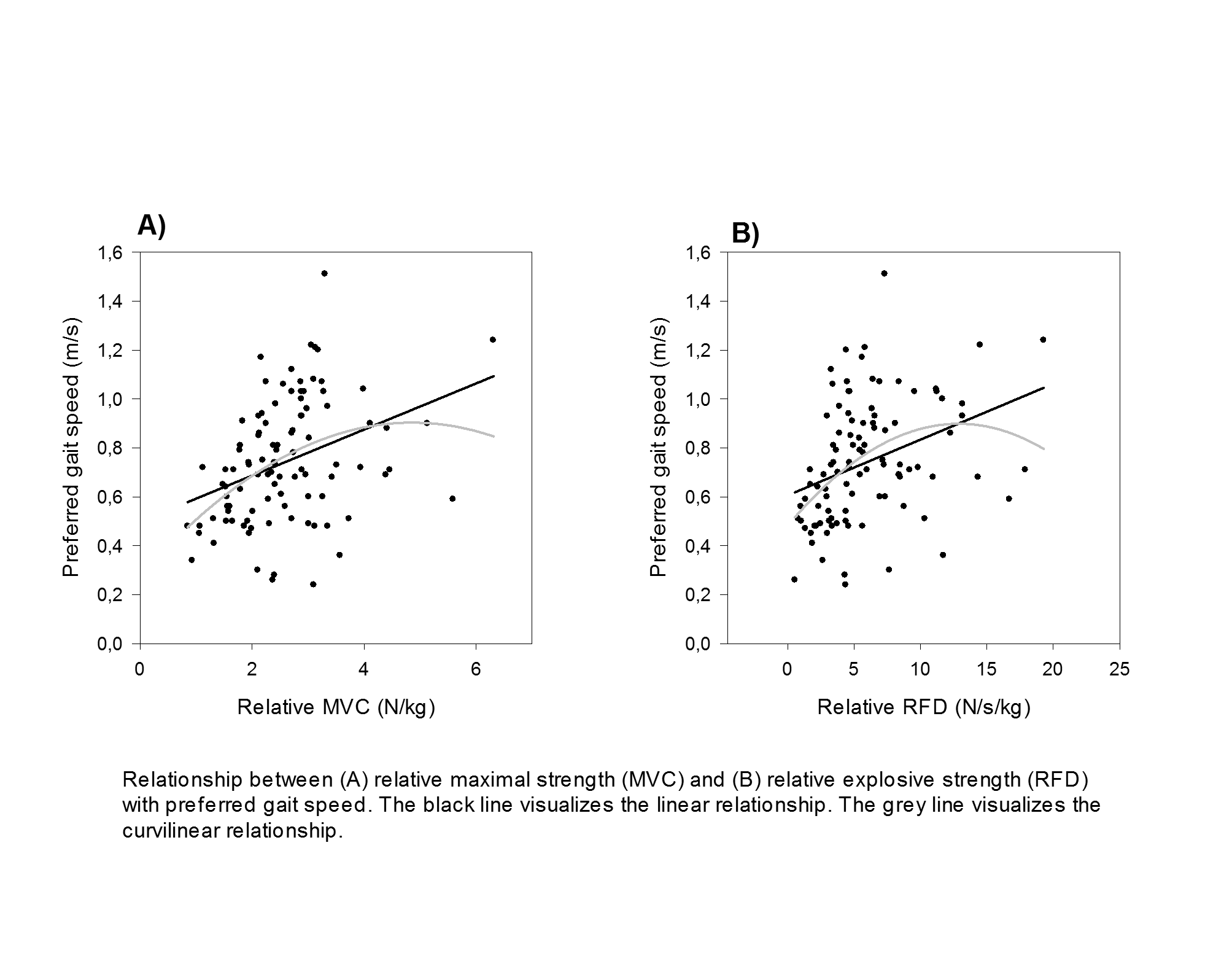

Supplement: Supplementary file 5 [file Image_3.tif]

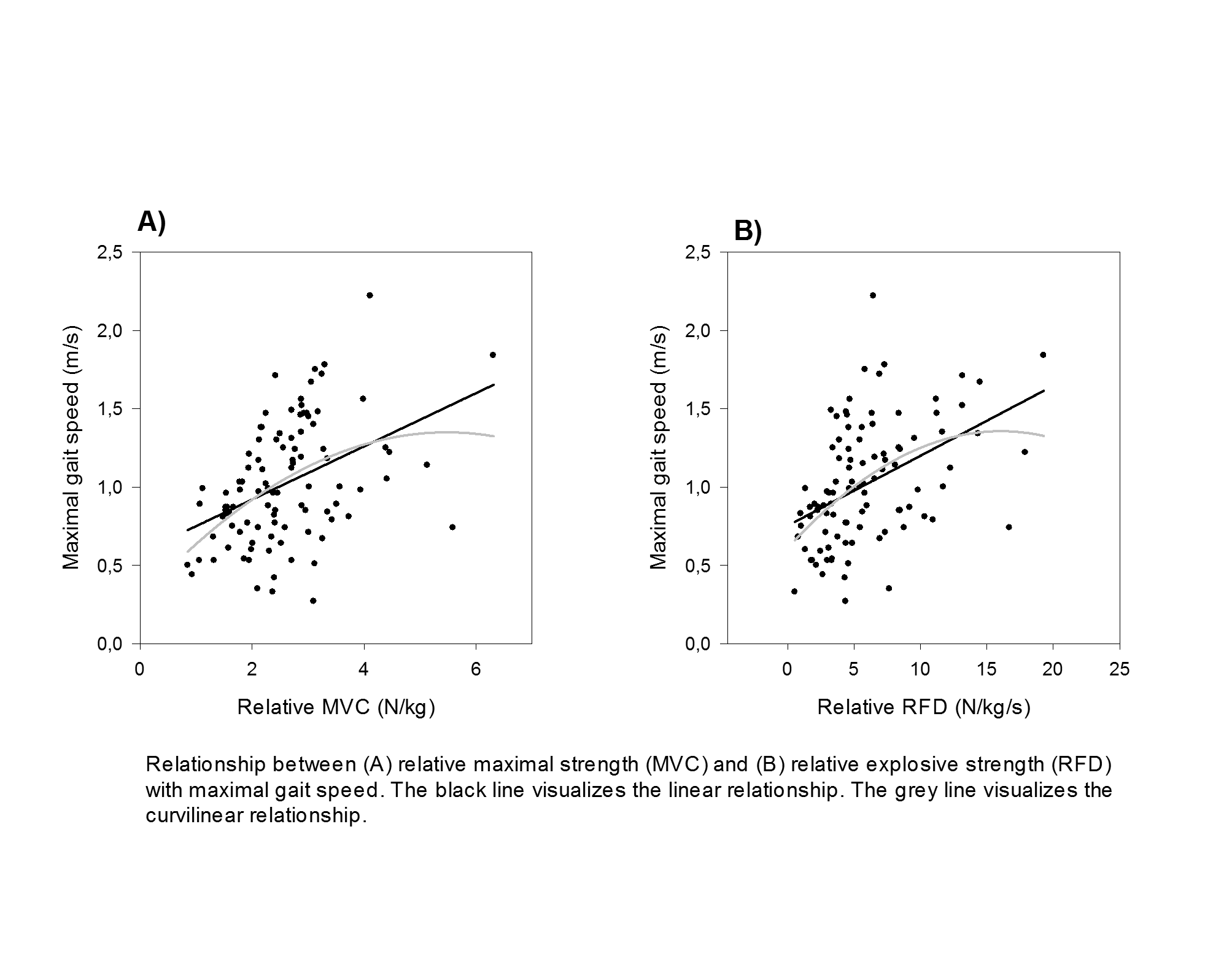

Supplement: Supplementary file 6 [file Image_4.tif]
